# Supplementary material for: Understanding the mechanism of catalytic fast pyrolysis by unveiling reactive intermediates in heterogeneous catalysis
Source: Nat Commun. 2017 Jun 29;8:15946. doi: 10.1038/ncomms15946 (PMC5493764; doi:10.1038/ncomms15946)
Supplement: Supplementary Information [file ncomms15946-s1.pdf]

Type of file: PDF
Size of file: 0 KB
Title of file for HTML: Supplementary Information
Description: Supplementary Figures, Supplementary Tables, Supplementary Notes, Supplementary Methods and Supplementary References

Type of file: PDF
Size of file: 0 KB
Title of file for HTML: Peer Review File
Description:

Supplementary Methods:

The PEPICO endstation (Supplementary Fig. 1, left), the measurement techniques and the VUV beamline at Paul Scherrer Institute was described elsewhere.[1-3] The catalytic reactor used in our experiments applies the Temporal Analysis of Products (TAP) approach, which was introduced by Gleaves et al.[4] The design and performance is similar to the reactor published by Leppelt et al. and is depicted in Supplementary Figure S1 (right).[5] In brief, the TAP flange consists of a base plate, which is mounted on a differentially pumped gate valve. The valve allows for roughing the reactor before it is opened to the main high vacuum ionization chamber of the iPEPICO endstation. The quartz glass tube (Supplementary Figure S1Fig. 1) is connected to the valve manifold using Ultra-Torr® fittings, equipped with high-temperature Kalrez O-rings. Six cartridge heaters with a total power of 900 W heat the reactor and the temperature is measured directly at the outer surface of the reactor wall within the furnace by a type-K thermocouple. An external controller ensures temperature stability over several hours of operation. Three Gyger valves (SMLD 300 G, 100 µm orifice) are connected to the valve manifold enabling alternating pulse experiments as well as conditioning of the catalyst prior to use. Supplementary Figure S1Fig. 1 (lower part) shows pulse profiles of an argon pulse (blue line) travelling through an empty reactor at 400 °C. The guaiacol signal (red line) is also plotted, which does not decay as quickly as the argon signal, speaking for a chromatographic effect occurring at the wall of the quartz glass reactor. Upon applying the catalyst coating to the walls of the tube, the argon pulse shape does not change significantly; however, the guaiacol signal decreases by almost two orders of magnitude, attesting to significant adsorption on the catalyst surface.

A mixture of guaiacol and argon is pulsed in the reactor for 5–10 hours in a typical experiment. The guaiacol signal increases with time (time-on-stream), but no pulse structure can be extracted even at very low repetition rates of 0.1 Hz. Thus, the process is completely desorption limited, which is also mirrored by Supplementary Figure S1Fig. 3. Intermediates and reaction products are only observed after several minutes/hours of constant application of guaiacol (2.5 Hz repetition rate), making an analysis of the pulse profiles redundant. Some products, such as catechol, are only desorbed at long time-on-streams. The overall residence time of the reactant, products and intermediates on the catalyst greatly exceeds the minimum time resolution (20 ms, fwhm) of our TAP reactor.

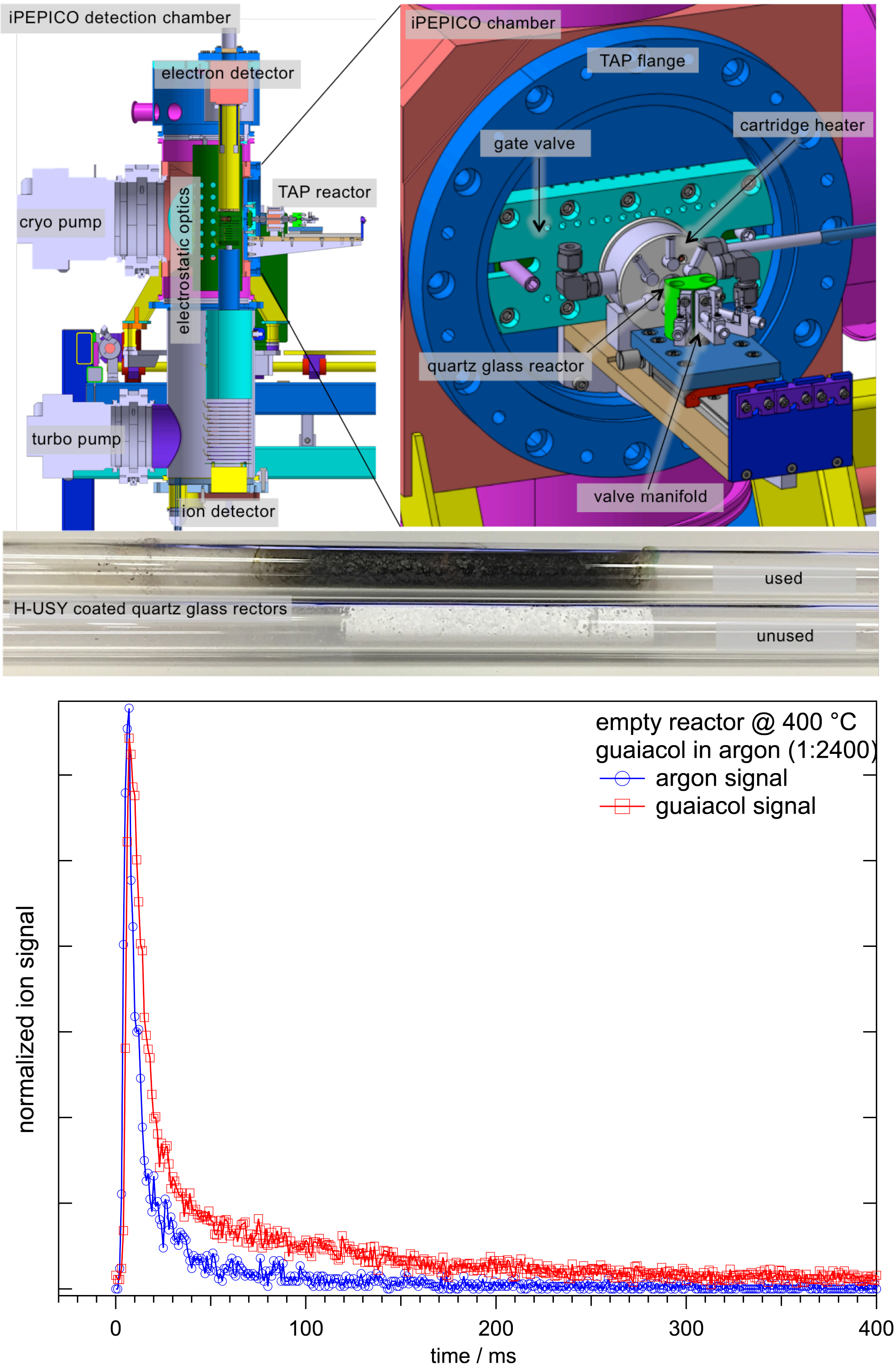

Supplementary Figure 1. Upper part: Representation of the TAP reactor setup connected to the iPEPICO endstation located at the Swiss Light Source at Paul Scherrer Institute. A detailed view of the reactor manifold is depicted right hand side. Lower part: Typical pulse profiles through an empty reactor at 400 °C.

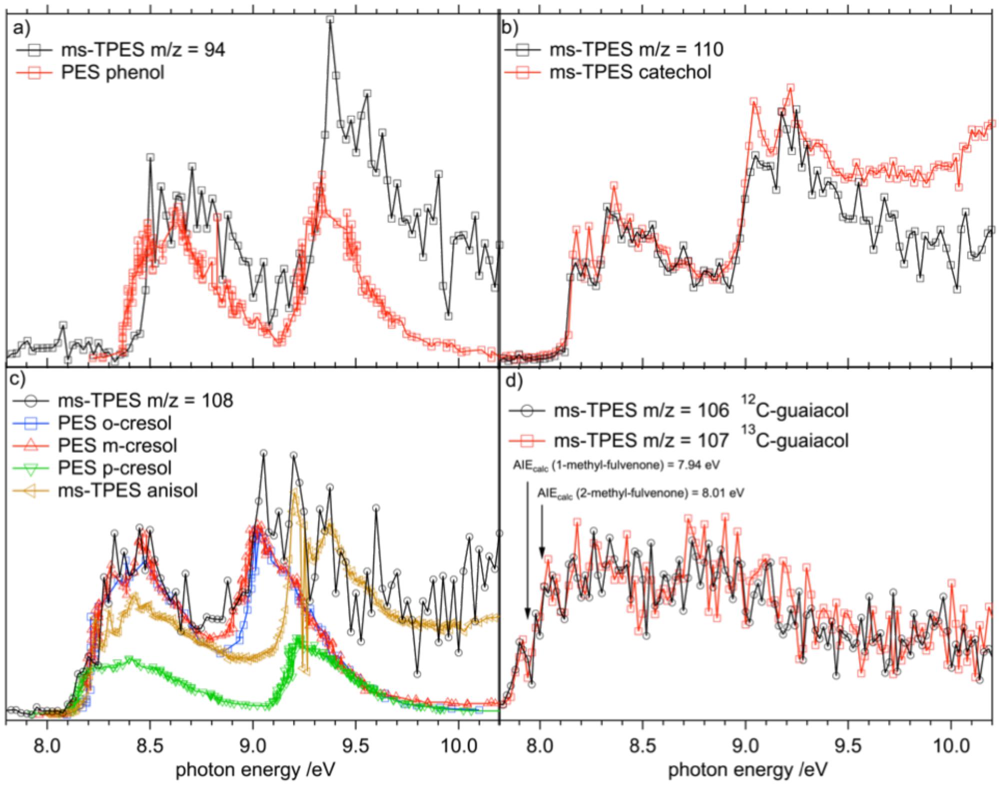

Supplementary Figure 2. ms-TPE spectra of phenol (*m*/*z* = 94), catechol (*m*/*z* = 110), cresols (*m*/*z* = 108), and methyl-fulvenones (*m*/*z* = 106), along with literature reference spectra of the pure compounds.[6,7] For the latter species, no reference spectrum is available, and a FC simulation was not possible due to the pseudo-rotation of the methyl group upon ionization, leading to large amplitude motions. However, CBS-QB3 calculations revealed that two isomers contribute to the *m*/*z* = 106 ms-TPES, namely 1-, and 2-methyl-fulvenone. ^{13}C-labeling shifts the spectrum by one mass-to-charge unit, ruling out contributions from xylenes, which have the same unit molecular mass in the non-labeled experiments. The *m*/*z* = 108 ms-TPES consists of four different isomers of the composition C_8H_8O. Besides the three cresols (*o*-, *p*-, and *m*-methyl-phenol), the contribution of anisol (methoxy benzene) is revealed by the intense transition at 9.25 eV belonging to an electronically excited cation state.

Supplementary Note 1: Number of molecules per pulse, weighted hourly space velocity (W/F), and time-on-stream curves of guaiacol over H-USY

We have connected the pulsed valves to a gas mixing unit consisting of a gas tank, valves and tubing. The pressure in the gas tank changes during the experiment and we determine the number of molecules in view of the pressure change using the ideal gas law and the total tank volume:

$\Delta pV = \Delta NkT$

The number of molecules per gas pulse is obtained by dividing with the number of pulses, and was typically in the order of 1×10^{16} molecules per pulse. Taking into account the vapor pressure of guaiacol, 0.15 mbar at 25 °C, and assuming complete saturation of argon at ca. 400 mbar absolute pressure, a dilution of 1:2400 is achieved, giving 5×10^{12} guaiacol molecules per pulse. The weighted hourly space velocity (W/F) is defined as the quotient of the mass flow rate (W) of the reactants divided by the mass of the catalyst in the reactor. A mass flow rate (W) can be calculated at 2.5 Hz repetition rate yielding, depending of loading of the reactor (0.5-3 mg, F), a W/F of between 0.01 and 0.003 h.

Supplementary Fig. 3 shows the time profile of the desorbed species as a function of time or amount of guaiacol feed into the reactor. Comparing the guaiacol signal strength of an empty versus H-USY-coated hot reactor, the signal drops by almost two orders of magnitude in the latter case, meaning that almost all guaiacol is adsorbed on the catalyst once the feed starts. At the beginning of the feed, the guaiacol signal remains fairly constant indicating a constant fraction of the pulse that passes through the reactor without being adsorbed on the catalyst surface. However, after around 150 min (> 25 µg guaiacol feed), the signal increases again, due to saturation of the catalyst surface by guaiacol or adsorbed reaction intermediates and products. In addition, deactivation of the catalyst can also contribute to the increasing guaiacol signal. Indeed, after 5–10 h time-on-stream, the catalyst turns strongly brownish or black indicating coking. Coking is known as one of the major deactivation mechanisms of the catalyst.[8,9] However, it also has positive effects, as discussed below.

Phenols and methyl-phenols, including anisole, are the major pyrolysis conversion products, and start to desorb after around 100 min (20 µg guaiacol feed), similar to the unreacted guaiacol. Since transalkylation often occurs under presence of methoxy groups it is obvious that methyl-phenols (and anisole) are yielded by methylation of phenol.[10] More apolar, and therefore less strongly bound non-oxygenated species, such as cyclopentadiene (*c*-C_5H_6), methyl-cyclopentadienes, fulvene and benzene, desorb earlier from the reactive surface of the zeolite catalyst compared to the major polar products

(Supplementary Fig. 3, lower trace). The two ketenes, ethenone ($H_2C=C=O$) and 6-fulvenone ($c\text{-}C_5H_4=C=O$) appear simultaneously with the non-oxygenated species. Catechol (m/z = 110) is the most strongly adsorbed species on the surface among the ones that can escape it, which may be explained by presence of two OH groups, both of which can form hydrogen bonds on the acid sites of the catalyst. Xylenols (*m*/*z* = 122) appear also at the same time as the major products, but at significantly smaller yields. The fact that similar molecules (phenol & cresols and cyclopentadiene & methyl-cyclopentadienes) with similar adsorption enthalpies on H-USY, desorb at almost the same time, also point to a desorption limited process. Thus, the TOS scans may not show the appearance and disappearance of intermediates exactly, and do not reflect the reaction pathways directly. However, they give information on the state and performance of the catalyst as a function of time.

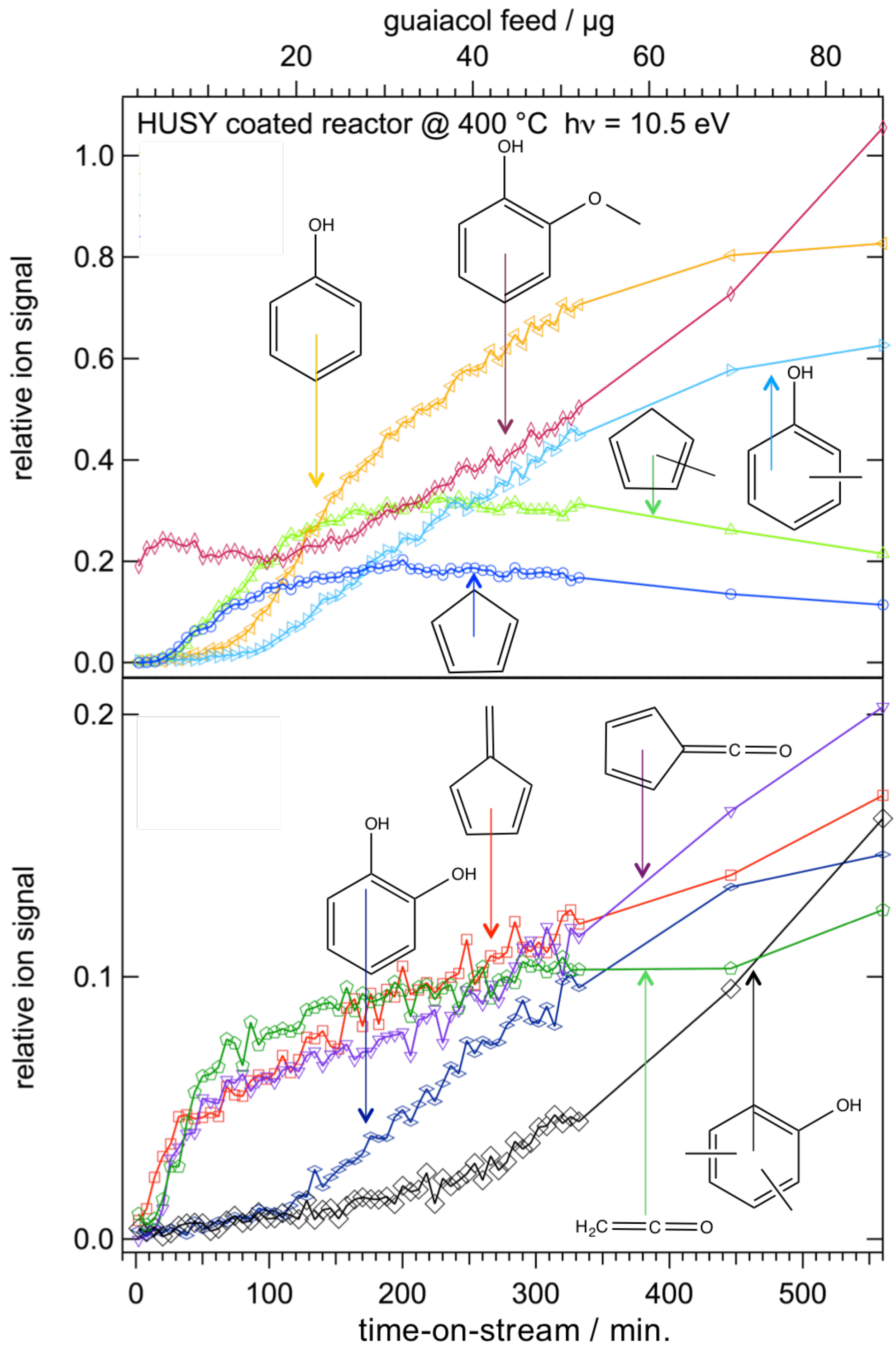

Supplementary Figure 3. Time profiles of the guaiacol H-USY reaction as a function of time or guaiacol feed: (methyl)-cylclopentadienes, fulvenone, ethenone and fulvene are desorbed first. The major products: cresols, anisols, and phenol appear significantly later (or at higher guaiacol concentration on the catalyst).

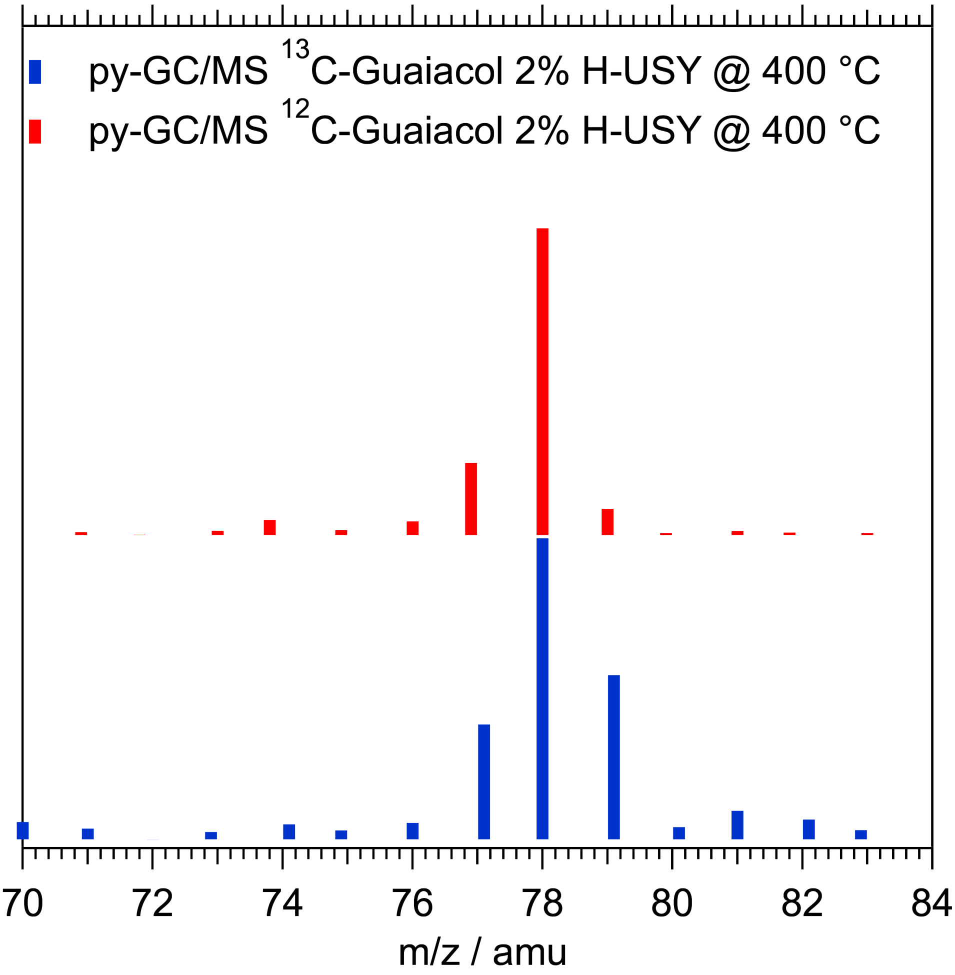

Supplementary Figure 4. Comparison C_6H_6 in py-GC/MS with ^{12}C- and ^{13}C-labeled guaiacol on H-USY: Upon ^{13}C-labeling, the benzene peak does not clearly shift by one *m/z* unit. It rather shows an increasing ^{13}C contribution, also indicative of the two pathways to yield benzene in accordance with the py-iPEPICO results.

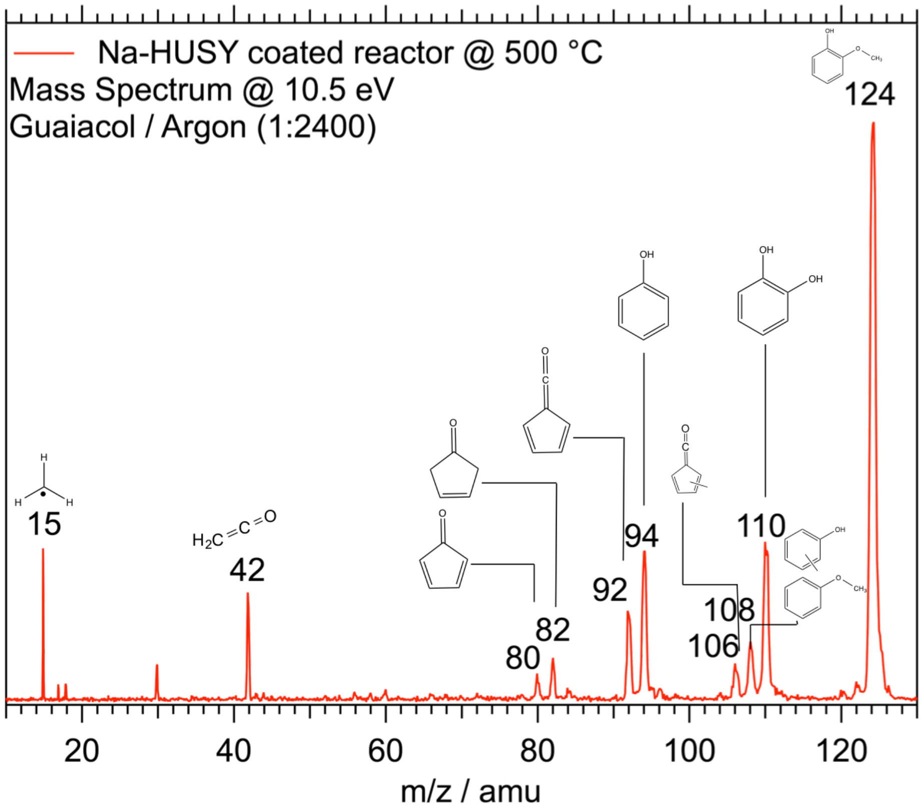

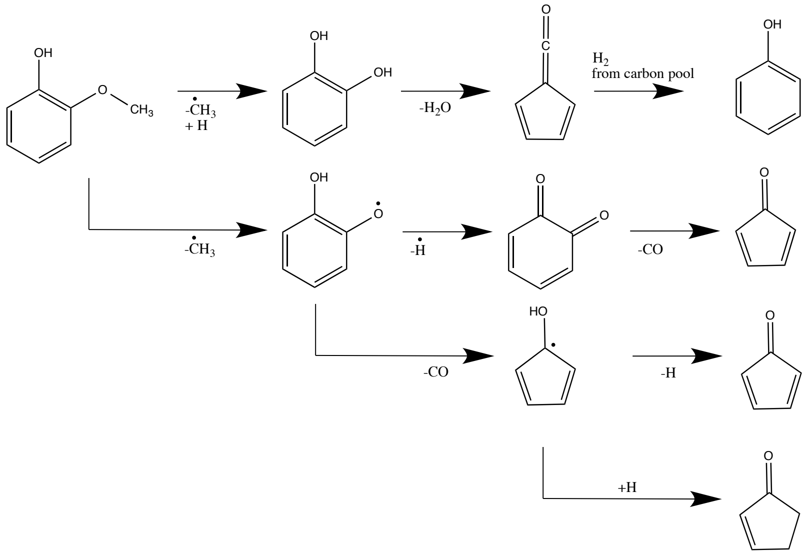

Supplementary Figure 5. Mass spectrum taken at 10.5 eV using Na-USY @ 500°C, with ^{12}C-guaiacol as precursor. Apart from the much lower conversion compared to H-USY, the appearance of methyl radical (*m*/*z* = 15) along with cyclopentadienone (*m*/*z* = 80), both assigned based on their ms-TPES, is eye catching. Therefore, another radical pathway exists besides the acid catalyzed pathway driving transmethylation reactions and dehydration of catechol, as discussed in the main manuscript. This new pathway yields reactive species, such as methyl radicals. The reaction probably proceeds as follows. After methyl abstraction, a hydrogen addition occurs yielding catechol, which can decompose in a Lewis acid catalyzed way yielding fulvenone (*m*/*z* = 92) and later phenol (*m*/*z* = 94). In addition, the hydroxyl-phenoxy radical (not observed) can lose hydrogen and decabonylate to cyclopentadienone (*m*/*z* = 80). Hydrogen addition on the other hand may be responsible for cyclopentenone formation (*m*/*z* = 82), which was also observed during the Na-USY experiments. The reaction steps are summarized in the lower part of the figure.

Supplementary Table 1. Summary of the detected species in both setups. Adiabatic ionization energies IEs (see references), Franck Condon (FC) simulations or literature PES (see references) were used to identify the species in the py-iPEPICO setup.

			detection experiment		
**m/z**	isomer/compound	IE / eV	py-GC/MS (selectivity)	py-iPEPICO	^{13}C labeled
**28**	CO	14.014 [11]	✓	✓	✗
**42**	Ethenone $H_2C{=}C{=}O$	9.6191 [12]	✗	✓	(✓)
**66**	Cyclopentadiene, c-C_5H_6	8.57 [13]	✗	✓	✗
**78**	Benzene, c-C_6H_6	9.24384 [14]	✓	✓	(✓)
	Fulvene, c-$C_5H_4{=}CH_2$	8.36 [15]	✗	✓	✓
**80**	1-methyl-cyclopentadiene, c-$C_5H_5(CH_3)$	8.14 [16]	✗	✓	✓
	2-methyl-cyclopentadiene, c-$C_5H_5(CH_3)$	8.28 [16]	✗	✓	✓
	5-methyl-cyclopentadiene, c-$C_5H_5(CH_3)$	8.50 [16]	✗	✓	✓
**92**	Toluene, C_6H_5-CH_3	8.828 [17]	✓ (6%)	✓	✓
	6-fulvenone, c-$C_5H_4{=}C{=}O$	8.26 [this work]	✗	✓	✗
**94**	phenol	8.49 [18]	✓ (11%)	✓	✗
**106**	o-, m-, p-xylene (C_8H_{10})	8.44-8.56 [19]	✓ (5%)	(✓)	✓
	methyl-fulvenones, c-C_5H_3(CH3)=C=O	7.94 & 8.01 [this work]	✗	✓	✓
**108**	Ansiol, methoxybenzene, C_7H_8O	8.39 [20]	✓ (1%)	✓	✓
	o-cresol, 2-methylphenol, C_7H_8O	8.48 [20]	✓	✓	✓
	m-cresol, 3-methylphenol, C_7H_8O	8.52 [20]	✓ (3%)	✓	✓
	p-cresol, 4-methylphenol, C_7H_8O	8.38 [20]	✓	✓	✓
**110**	Catechol, 1,2-benzenediol, $C_6H_6O_2$	8.15 [21]	✓	✓	✗
**120**	Trimethyl-benzenes, C_9H_{12}	8.27-8.42 [19]	✓ (7%)	✗	✓
**122**	Dimethyl-phenols, $C_8H_{10}O$	8.0-8.26 [20,22]	✓ (23%)	✓	✓
**124**	Guaiacol, 2-methoxyphenol, $C_7H_8O_2$	7.93 [this work]	✓	✓	✓
	Methyl-catechol, $C_7H_8O_2$	?	✓ (11%)	✗	✓
**136**	Trimethylphenol, $C_9H_{12}O$	8.0 [23]	✓ (1%)	(✓)	(✓)
**138**	Methoxymethylphenol, $C_8H_{10}O_2$	?	✓ (9%)	(✓)	(✓)

Supplementary Note 2: Additional Experiments: py-GC/MS with cyclopentadiene

Due to the presence of stable intermediates such as cyclopentadiene and methyl-cyclopentadiene in the py-iPEPICO setup, we have carried out a set of experiments to explain, why they evade detection in the py-GC/MS setup.

Firstly, the H-USY catalyst was loaded with guaiacol in the batch-type reactor and the helium flow rates through the reactor into the GC/MS were varied between 0.8 and 4.0 sccm. In addition, we have measured at several oven temperatures of the transfer line (175, 250 and 325 °C). Both changes did not lead to an enhancement of any five-membered ring species, but the conversion and selectivity remains identical.

Secondly, freshly prepared cyclopentadiene (from dicyclopentadiene at 180 °C) was directly injected onto the glass wool. Using the same conditions as in the experiments with H-USY and guaiacol, cyclopentadiene could be observed in the GC/MS system, without any further reaction. Only traces of the dimer, dicyclopentadiene, were detected.

Upon introducing H-USY catalyst, the amount of desorbed CP dropped by several orders of magnitude compared to the previous experiment, indicating strong adsorption on the catalyst. Besides benzene, toluene, xylenes, indane and naphthalene only trace amounts of cyclopentene, cyclopentane and methyl-cyclopentane have been detected, but the overall yield of CP is small. Increasing the concentration of CP on the catalyst did not lead to a higher CP signal, but to significant increase in the coke concentration, as indicated by color change of the used zeolite from brownish to black.

Thirdly, we wanted to investigate the lifetime and stability of cyclopentadiene in the reactive and catalytic environment. Thus, we have applied mixtures of guaiacol and cyclopentadiene (4:1) on the catalyst. Here, cyclopentadiene completely evades detection and we have found only stable intermediates and reaction products as compared to the experiments without CP. This observation proves that CP is stable enough to be transferred in the gas phase and to be detected by GC/MS, however, as soon as other reactants are present in the reaction mixture CP rapidly reacts further on the catalyst than being desorbed from the surface. The results confirm the difference in the two analytical setups: the py-iPEPICO has a very short residence time in the reactor and the catalyst, while the batch-type py-GC/MS has an increased residence time in the reactor (and on the catalyst) by several orders of magnitudes (20 seconds compared to a few µsec).

Supplementary Note 3: Synthesis and Catalyst Characterization

The ^{13}C-labelled guaiacol was synthesized according to Lee et al. with ^{13}C-CH_3I and catechol (both from Sigma-Aldrich), base catalyzed with Cs_2CO_3 (Acros) in acetonitril for 5 h at 35 °C. The yield was 42 wt.% after purification.[24]

Commercial H-USY (Zeochem) has a Si/Al ratio of 7 and a total number of acid sites of 1.21 mmol/g determined by ammonia desorption from the NH_4^+ form. USY has a BET surface area of 530 m^2g^{-1}, which is assumed to be maintained after ion exchange. Na-USY was obtained by an ion exchange reaction performed three times using $NaNO_3$. The USY-zeolites (Zeochem) were calcined at 550 °C for 5 h prior to use.

Cyclopentadiene was synthesized from the dicyclopentadiene by pyrolysis in an oil bath at 175 °C. The cyclopentadiene was distilled (38-42 °C, 1013 mbar) from the reaction mixture, according to the literature.[25,26]

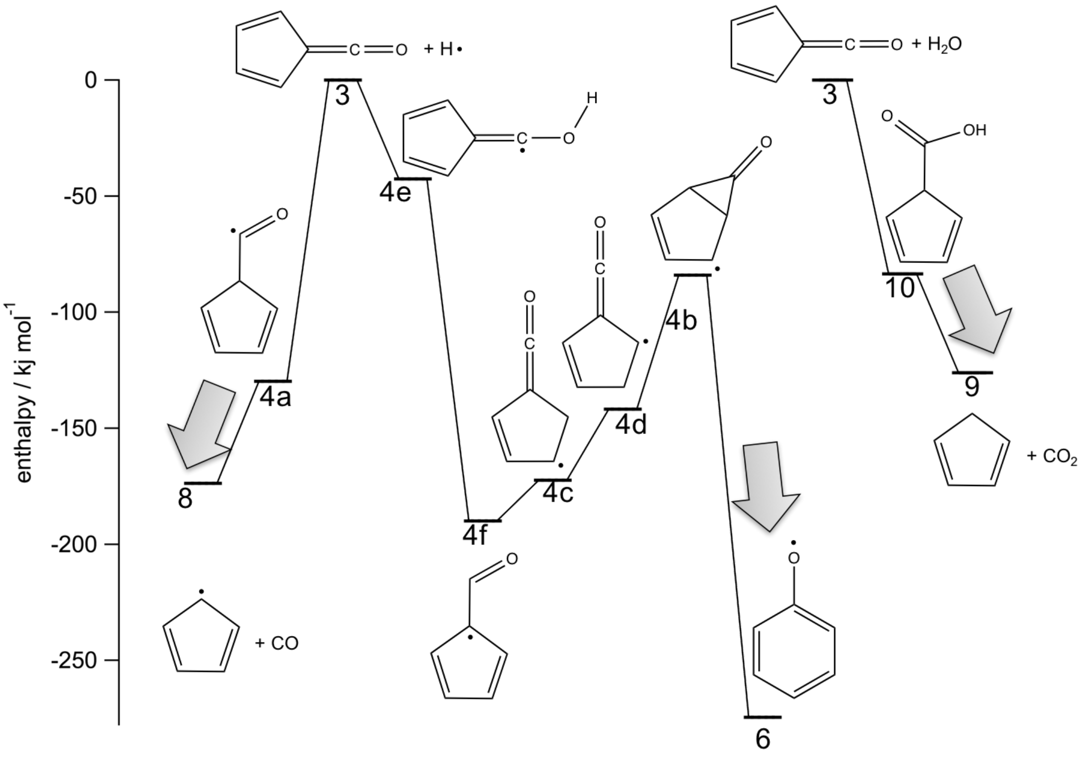

Supplementary Figure 6. Enthalpy diagram of the fulvenone hydrogen radical addition. After hydrogenation six isomers (**1**-**6**) of the composition C_6H_5O can equilibrate quickly and further stabilization can occur to yield either cyclopentadienyl (**3** to **8**) or phenoxy radicals (**6** to **7**). In addition the hydrolysis pathway of ketene to yield carbon dioxide and *c*-C_5H_6 is also depicted. Grey arrows denote effective reaction channels.

Supplementary References:

1 Bodi, A. *et al.* Imaging photoelectron photoion coincidence spectroscopy with velocity focusing electron optics. *Rev. Sci. Instrum.* **80**, 034101, (2009).

2 Bodi, A., Sztáray, B., Baer, T., Johnson, M. & Gerber, T. Data acquisition schemes for continuous two-particle time-of-flight coincidence experiments. *Rev. Sci. Instrum.* **78**, 084102, (2007).

3 Johnson, M., Bodi, A., Schulz, L. & Gerber, T. Vacuum ultraviolet beamline at the swiss light source for chemical dynamics studies. *Nucl. Instrum. Meth. A* **610**, 597-603, (2009).

4 Gleaves, J. T., Ebner, J. R. & Kuechler, T. C. *Catal. Rev. - Sci. Eng* **30**, 49, (1988).

5 Leppelt, R. *et al.* Design and characterization of a temporal analysis of products reactor. *Rev. Sci. Instrum.* **78**, 104103, (2007).

6 Tsunetoshi, K. & Saburo, N. Photoelectron spectra of substituted benzenes. *Bull. Chem. Soc. Jpn.* **47**, 2563-2572, (1974).

7 Kimura, K., Katsumata, S., Achiba, Y., Yamazaki, T. & Iwata, S. *Handbook of hei photoelectron spectra of fundamental organic molecules.* (Japan Scientific Socienties Press, Tokyo, 1981).

8 Ma, Z. & van Bokhoven, J. A. Deactivation and regeneration of h-usy zeolite during lignin catalytic fast pyrolysis. *ChemCatChem* **4**, 2036-2044, (2012).

9 Mukarakate, C. *et al.* Real-time monitoring of the deactivation of hzsm-5 during upgrading of pine pyrolysis vapors. *Green Chem.* **16**, 1444-1461, (2014).

10 Thilakaratne, R., Tessonnier, J.-P. & Brown, R. C. Conversion of methoxy and hydroxyl functionalities of phenolic monomers over zeolites. *Green Chem.* **18**, 2231-2239, (2016).

11 Erman, P. *et al.* Direct determination of the ionization potential of co by resonantly enhanced multiphoton ionization mass spectroscopy. *Chem. Phys. Lett.* **215**, 173-178, (1993).

12 Niu, B., Bai, Y. & Shirley, D. A. High resolution photoelectron spectroscopy and femtosecond intramolecular dynamics of h2cco+ and d2cco+. *J. Chem. Phys.* **99**, 2520-2532, (1993).

13 Derrick, P. J., Åsbrink, L., Edqvist, O., Jonsson, B. Ö. & Lindholm, E. Rydberg series in small molecules. *Int. J. Mass Spectrom. Ion Physics* **6**, 203-215, (1971).

14 Nemeth, G. I., Selzle, H. L. & Schlag, E. W. Magnetic zeke experiments with mass analysis. *Chem. Phys. Lett.* **215**, 151-155, (1993).

15 Bieri, G., Burger, F., Heilbronner, E. & Maier, J. P. Valence ionization energies of hydrocarbons. *Helv. Chim. Acta* **60**, 2213-2233, (1977).

16 Bierkandt, T., Hemberger, P., Oßwald, P., Köhler, M. & Kasper, T. Insights in m-xylene decomposition under fuel-rich conditions by imaging photoelectron photoion coincidence spectroscopy. *Proc. Combust. Inst.* **36**, 1223-1232, (2017).

17 Lu, K. T., Eiden, G. C. & Weisshaar, J. C. Toluene cation: Nearly free rotation of the methyl group. *J. Phys. Chem.* **96**, 9742-9748, (1992).

18 Fuke, K. *et al.* Multiphoton ionization photoelectron spectroscopy and two-color multiphoton ionization threshold spectroscopy on the hydrogen bonded phenol and 7-azaindole in a supersonic jet. *Chem. Phys. Lett.* **108**, 179-184, (1984).

19 Howell, J. O. *et al.* Electron transfer from aromatic hydrocarbons and their .Pi.-complexes with metals. Comparison of the standard oxidation potentials and vertical ionization potentials. *J. Am. Chem. Soc.* **106**, 3968-3976, (1984).
20 Kobayashi, T. & Nagakura, S. Photoelectron spectra of substituted benzenes. *Bull. Chem. Soc. Jpn.* **47**, 2563-2572, (1974).
21 Palmer, M. H., Moyes, W., Speirs, M. & Ridyard, J. N. A. The electronic structure of substituted benzenes; ab initio calculations and photoelectron spectra for phenol, the methyl- and fluoro-derivatives, and the dihydroxybenzenes. *J. Mol. Struct.* **52**, 293-307, (1979).
22 Russell, D. H., Freiser, B. S., McBay, E. H. & Canada, D. C. The structure of decomposing [c7h7o]+ ions: Benzyl versus tropylium ion structures. *Org. Mass Spectrom.* **18**, 474-485, (1983).
23 Cetinkaya, B., Lappert, M. F. & Suffolk, R. J. Photoelectron spectra of some sterically hindered phenols and related compounds. *J. Chem. Res. Synop.*, 316, (1983).
24 Lee, J. C., Yuk, J. Y. & Cho, S. H. Facile synthesis of alkyl phenyl ethers using cesium carbonate. *Synth. Commun.* **25**, 1367-1370, (1995).
25 Moffett, R. B. *Org. Synth., Coll. Vol. IV*, 238-241, (1963).
26 Partridge, J. J., Chadha, N. K. & Uskokovic, M. R. *Org. Synth., Coll. Vol. Vll*, 339-345, (1990).
